# Inflammatory stress on induced neurons carrying Parkinson's disease‐associated LRRK2 Mutations

**DOI:** 10.1002/alz70855_107448

**Published:** 2025-12-26

**Authors:** Jose Torrellas, Aravindraja Chairmandurai, Adamantios Mamais, Matthew J LaVoie, Paramita Chakrabarty

**Affiliations:** ^1^ University of Florida, Gainesville, FL, USA; ^2^ University of Florida College of Medicine, Gainesville, FL, USA

## Abstract

**Background:**

Mutations in the leucine‐rich repeat kinase 2 (*LRRK2*) gene, particularly the *G2019S* and *R1441C* variants, are the most prevalent genetic determinants of Parkinson's disease (PD). *LRRK2* is increasingly recognized for its roles in immune signaling, with studies suggesting that interferon‐gamma (IFNγ), a master immune regulator, may modulate its activity. Based on these findings, we hypothesize a synergistic interaction between *LRRK2* mutations and IFNγ.

**Method:**

To explore this hypothesis, we utilized neurons derived from human induced pluripotent stem cells (iPSCs) harboring homozygous *G2019S* or *R1441C LRRK2* mutations, with isogenic wildtype (WT) controls. After differentiation into neurons, cells were treated with recombinant human IFNγ or control media on day 17 of culture and harvested after 6 hours of treatment. Six experimental conditions were analyzed: (WT + IFNγ, WT + Control, G2019S + IFNγ, G2019S + Control, R1441C + IFNγ, R1441C + Control) We analyzed these experiments using immunoblotting and immunofluorescence methods to assess interferon‐pathways, tau phosphorylation, and LRRK2 substrate engagement.

**Result:**

Using a dose‐dependent paradigm, we optimized the minimum IFNγ dose to achieve maximal target engagement (STAT1 and phosphorylated‐STAT1, pSTAT1). All three genotypes responded to IFNγ, producing equivalent levels of pSTAT1, confirming activation of the JAK‐STAT pathway. We found that at baseline, levels of pSer202 tau (CP13) levels were moderately increased in both G2019S‐ and R1441C‐LRRK2 relative to WT‐LRRK2. IFNγ treatment increased CP13 levels in both G2019S‐ and R1441C‐LRRK2, with the CP13 induction higher in G2019S‐LRRK2 while WT‐LRRK2 neurons did not show elevated CP13 following IFNγ treatment. IFNγ treatment phosphorylated Threonine73 on Rab10 (a LRRK2 substrate) more efficiently in G2019S‐LRRK2 neurons compared to R1441C‐LRRK2. We did not observe a genotype‐dependent effect of IFNγ on phosphorylation of Ser106 on Rab12, another LRRK2 substrate.

**Conclusion:**

Our data indicates a LRRK2 genotype related response of LRRK2 neurons to IFNγ stimulus. This data lays the groundwork for future studies to dissect mechanisms of neuronal vulnerability in PD and the interplay between *LRRK2* mutations and disease pathways.

